# Multi-omics approach to identifying isoform variants as therapeutic targets in cancer patients

**DOI:** 10.3389/fonc.2022.1051487

**Published:** 2022-11-24

**Authors:** Timothy I. Shaw, Bi Zhao, Yuxin Li, Hong Wang, Liang Wang, Brandon Manley, Paul A. Stewart, Aleksandra Karolak

**Affiliations:** ^1^ Department of Biostatistics and Bioinformatics, H. Lee Moffitt Cancer Center and Research Institute, Tampa, FL, United States; ^2^ Department of Machine Learning, H. Lee Moffitt Cancer Center and Research Institute, Tampa, FL, United States; ^3^ Center for Proteomics and Metabolomics, St. Jude Children’s Research Hospital, Memphis, TN, United States; ^4^ Department of Tumor Biology, H. Lee Moffitt Cancer Center and Research Institute, Tampa, FL, United States; ^5^ Department of Genitourinary Oncology, H. Lee Moffitt Cancer Center and Research Institute, Tampa, FL, United States

**Keywords:** proteomics, transcriptomics, alternative splicing, biomarker, therapeutic discovery

## Abstract

Cancer-specific alternatively spliced events (ASE) play a role in cancer pathogenesis and can be targeted by immunotherapy, oligonucleotide therapy, and small molecule inhibition. However, identifying actionable ASE targets remains challenging due to the uncertainty of its protein product, structure impact, and proteoform (protein isoform) function. Here we argue that an integrated multi-omics profiling strategy can overcome these challenges, allowing us to mine this untapped source of targets for therapeutic development. In this review, we will provide an overview of current multi-omics strategies in characterizing ASEs by utilizing the transcriptome, proteome, and state-of-art algorithms for protein structure prediction. We will discuss limitations and knowledge gaps associated with each technology and informatics analytics. Finally, we will discuss future directions that will enable the full integration of multi-omics data for ASE target discovery.

## Introduction

**Figure 1:**
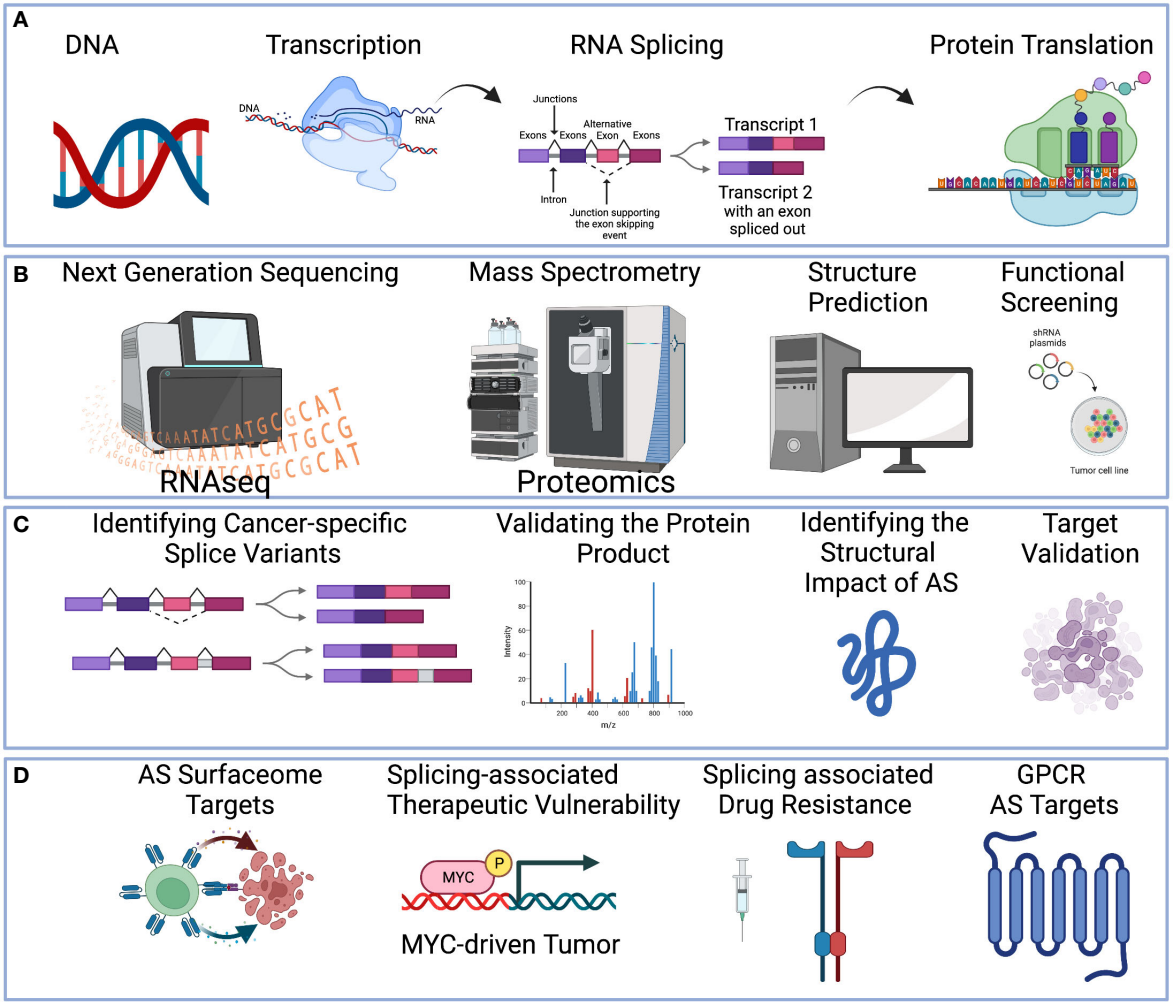
A holistic multi-omics approach to understanding therapeutic targets in cancer. **(A)** Central dogma of DNA-to-RNA followed by RNA splicing and protein translation. **(B)** Multi-omics technologies can be applied to study alternative splicing events, including High-throughput sequencing to profile the RNA transcript (Left-Most). Mass spectrometry technologies to profile the protein (Middle-Left). Protein structure can be predicted (Middle-Right). Functional screening such as siRNA or CRISPR can be applied (Right-Most). **(C)** RNA sequencing technologies can reveal the alternative splicing pattern (Left-Most). Mass spectrometry technologies can validate the protein product (Middle-Left). Structure prediction can identify the impact of alternative splicing (Middle-Right). Functional screening with oligonucleotide or CRISPR to screen for the functional effect of the target (Right-Most). **(D)** Examples of therapeutic strategies to target alternative splicing include splicing-derived neoantigens for immunotherapy, splicing-associated therapeutic vulnerability, splicing factor inhibitors, and small molecular inhibition to overcome drug resistance. Created with BioRender.com.

Alternative splicing is an integral transcriptional process that enables the production of multiple isoforms from a single gene (1, 2). The process is guided by the combinatorial inclusion and exclusion of exons or intron flanking regions (**Figure 1A**). Several pan-cancer studies of splicing-associated variants have suggested that cancer-specific isoform switches are part of the oncogenic process and contribute to the functional transformation of cancer cells (3–6). In cancer, these splicing-associated mutations can be either a trans-acting variant, which results in broad splicing change, or a cis-acting variant, which results in localized splicing change in a single gene (7). Examples of trans-acting variants include splicing factor (e.g., SF3B1, SRSF2, U2AF1, ZRSR2) mutations in both blood cancers (8–11) and solid tumors (12–15), and examples of the cis-acting splicing variants of cancer drivers include genome rearrangement, such as DUX4-ERG (16), point mutations in the splicing motifs of TP53 (17). Together, this splicing-associated genetic evidence suggests that alternative splicing events (ASEs) are important actors of the cancer oncogenic process (18) and can influence the cancer etiology in many situations.

Cancer-specific isoforms are RNA transcript isoforms exclusively expressed in cancer cells. Because they originate from somatic events (or from genes regulated by a cancer-cell epigenetic program), they are typically not expressed in normal tissue (19, 20). While these cancer isoform switches can be utilized as a biomarker for the clinical monitoring of adult malignancies (21, 22), several therapeutic strategies are available for targeting these alternative splicing events. Four major strategies include:

1. Chemotherapeutic inhibition of splicing factors, such as Sudemycin or other small molecules targeting SF3B1 (23–27).
2. Oligonucleotide therapy to activate or inhibit certain splicing events. Whereas splice-switching oligonucleotides (SSOs) can block access of the RNA splicing machinery to a splice site (28), a decoy RNA binding sequence can be used to inhibit the splicing factor activity by competing with its capacity for RNA binding (29).
3. Identification of oncogene with splicing-associated therapeutic vulnerability (See **Figure 1D**). Example of this includes MYC-driven cancers, such as neuroblastoma (30), lymphoma (31), and triple-negative breast cancer (32).
4. Immunotherapy targeting of neoantigen derived from cancer-specific ASEs (See **Figure 1D**). For example, neoantigens derived from these splicing alterations (7) can be used for mRNA vaccines (33), and surface antigens derived from cancer-specific isoforms can be targeted by CAR-T cell engineering, such as FN1 ED-B variant (34).

Given their therapeutic potential, identifying novel ASEs by high-throughput methods is critical to discovering new therapeutic targets for treating cancer patients.

Currently, the mining of therapeutic targets is often limited to short-read RNA sequencing analysis of gene-level data without isoform considerations. This can limit the discovery process to a small number of targets, not considering either the protein isoform or its functional consequences. Thus, we posit that integrative multi-omics profiling will be necessary for the effective prioritization of ASE events as therapeutic targets as well as to understand their role in reprogramming the molecular network for cancer drug resistance (**Figures 1B, C**). These technologies include:

1. Short-and-long-read RNA sequencing for discovery and validation of novel transcript.
2. Mass spectrometry technologies to validate the protein isoform product.
3. Structure prediction of the protein isoform to identify small molecular binding sites.
4. Functional prediction of proteoform alterations and target validation by functional screening.

Through these technologies we will be able to prioritize and discover effective therapeutic strategies to target ASEs (**Figure 1D**). In this review, we will provide an overview of the different multi-omics technologies, bioinformatics algorithms, and existing knowledge gaps in the field of ASE therapeutic discovery.

## RNA-based technologies

Bulk RNA sequencing profiling is now a routine part of consortium projects and many clinical trials with ample opportunities for data mining. There are two library preparation protocols for RNA sequencing: 1) The polyadenylated (poly-A) protocol and 2) the Ribosome-depleted protocol (Total RNAseq). The poly-A method selects RNA transcripts with oligo (dT) primers (35), which effectively enriches mature RNA transcripts while eliminating transcripts lacking the poly-A RNA track added to the 3’ end, such as rRNA and most noncoding RNAs. Alternatively, the total RNAseq protocol performs ribosome depletion followed by sequencing, which retains both mature and immature transcripts without significant bias to the transcript direction.

There are several bioinformatics methods for analyzing short-read RNA sequencing data. For transcript-level bioinformatics analysis, the relative proportion of the full-length mRNA isoform can be estimated by RNAseq reads aligned to a given reference genome. The analysis can be divided into alignment-based algorithms and pseudo-alignment algorithms. Alignment-based algorithms include RSEM (36) and HISAT (37). RSEM aligns the reads directly onto the transcript sequence by considering the uncertainty of read mapping and then calculates a maximum likelihood abundance using the expectation-maximization algorithm (36). HISAT optimizes the indexing scheme by the Burrows-Wheeler transform algorithm and the Ferragina-Manzini index (37). More recently, pseudo alignment tools, such as Kallisto (38) and salmon (39), have gained in popularity. These algorithms perform alignment-free isoform quantification, providing orders of magnitude faster processing without sacrificing accuracy (40).

However, these strategies assume a complete reference of transcript isoform annotation, and missing transcripts may impact the final estimation (41). Another significant problem with transcript-level estimation is that RNA sequencing coverage bias can also impact the algorithmic performance (42). Therefore, alternative approaches are needed to complement the transcription estimation by examining local splicing changes in junction reads. Junctions are sequencing reads mapped to the genome reference that spans between two or more exons. Junction reads are critical in determining the exon usage pattern, and derived statistics can be applied to infer cancer-specific splicing events. One typical statistic for inferring these junction-associated changes is the percent spliced in (Ψ) score (43), which represents the exon inclusion percentage. Tools utilizing this strategy includes MISO (44), rMATS (45), MAJIQ (46), and SplAdder (47). Other algorithms for determining alternative splicing include LeafCutter (48) and Splicing Deficiency (49), which leverage the intron coverage to infer transcript usage and splicing dysregulation. These tools provide an additional alternative for detecting unannotated splicing events. Nonetheless, there are limitations to this type of approach. Ambiguity in the RNA mapping (such as repetitive regions) can impact junction coverage and result in a missed opportunity for identifying novel targetable isoforms. To overcome this junction coverage problem, long-read RNA sequencing or single-module RNA sequencing (50, 51) can be applied to capture the complete assembly of the transcript isoform and their alternative splice sites. There are two approaches for long-read sequencing: Pacific Biosciences’ single-molecular real-time sequencing and Oxford Nanopore Technologies’ nanopore sequencing. These technologies enable an average read length of > 30kb and can go up to 50kb in many situations. However, long-read sequencing technologies are largely limited with low sequencing depth (52). Fragmentation and pore-blocking may also occur in transcripts with high RNA modifications, resulting in a truncated transcript biased to the 3’ or 5’ end. Hence, a joint characterization of the short-read and long-read RNA sequencing may be an optimal strategy for characterizing novel transcripts. For example, long-read RNA sequencing data can guide the transcript assembly of the short-read RNA sequencing data. Moreover, this strategy can be enhanced by single-molecule barcoding, which simplifies the transcript assembly, facilitating new transcript identification and bulk RNA sequencing analyses (53, 54). Altogether, additional algorithm development to integrate both datasets will be necessary.

## Proteogenomic analysis

While the RNA-based sequencing approach can identify novel transcripts, a significant portion (~2000) of the novel RNA isoforms lack proof of generating a protein product (55, 56). Therefore, proteogenomics, a strategy of integrating RNA sequencing with mass spectrometry data, is needed to confirm these protein products. There are several software workflows for the preparation and analysis of proteogenomic data (57–59). Generally, these tools are used to build custom protein databases based on nucleotide sequences for use in proteomics experiments. For example, RNAseq can identify alternative splicing events at the transcript level that, when translated, do not appear in standard protein databases (*e.g.*, UniProt/SwissProt). Once assembled, these custom databases can be appended to standard (canonical protein) search databases, enabling the identification and quantification of non-standard peptides and proteins (*e.g.*, splice variants, single nucleotide polymorphisms, gene fusions).

Numerous efforts have been made to resolve isoform-level protein quantification in human tissues (60–63). For example, Lau et al. (62) used an RNAseq-guided method to generate a customized database of tissue-specific splicing junctions, which was searched against ~80 million public mass spectra. Hundreds of splicing events were identified for each tissue, including alternative 3’ and 5’ splice sites, mutually exclusive exons, intron retention, and skipped exons. In particular, the authors revealed significantly higher usage of alternative splicing events in the heart and testis. In another example, Kahles et al. describe analyses of alternate splicing in 8,705 cancer patients across 32 cancer types from The Cancer Genome Atlas (7). Of note, they focused on 63 patients from breast cancer and ovarian serous cystadenocarcinoma with proteomics data from the Clinical Proteomics Tumor Analysis Consortium (CPTAC) and validated neojunctions using proteomics data multiplexed with tandem mass tag (TMT) technology. Although they were able to validate the protein potential for over two-thirds (43/63) of the junction, more than a third of the junctions remained unvalidated. This is likely due to the limitation of the TMT-library technology, which requires pooling of samples into a single multiplexed experiment and leads to difficulties in identifying low-abundance proteins (64).

Despite high splicing diversity reported by transcriptomic studies (65, 66), in which >85% of genes have transcribed multiple non-trivial isoforms, proteogenomic studies tend to focus on a single protein isoform per gene or lack the resolution to identify alternative proteoforms (60, 61). This discrepancy can be partially explained by processes such as micro-RNA-based degradation  (67) or nonsense-mediated decay (68), but there are also technical challenges that restrict the detectability of isoforms. For example, trypsin is one of the most used enzymes for digesting proteins into peptides prior to LC-MS/MS. However, the ends of exons are enriched for trypsin digestion sites (i.e., lysine and arginine) (69), and the use of trypsin can digest peptides that would otherwise span the exon-exon junction that’s critical for making the splicing determination. Combining chymotrypsin with trypsin has partially alleviated this issue and resulted in increased junction peptide detection by >30% (69). Alternatively, the mass spectrometry machine can be programmed to monitor the specific mass range of the peptide supporting the proteoform. This targeted mass spectrometry strategy can enhance the proteomics identification of splice variants (70, 71). Moreover, a custom proteogenomic database built based on high-quality long-read sequencing transcripts can provide an improved custom database for mass spectrometry analysis (72, 73).

## Function and structural considerations of protein isoforms

A superior understanding of an exon’s structural properties and functional features of the encoded protein segment will be necessary to optimize therapeutic targets of alternatively spliced exons. Here, we review several computational methods and studies that attempted to perform functional characterization of protein isoforms. ASEs are often enriched in intrinsically disordered regions (IDRs) (74), regions lacking a well-defined tertiary structure. These disordered regions contain linear motifs and post-translational modification sites (PTMs), which are highly conserved in protein-protein interactions (74). IDRs are important in maintaining cellular functions (75), play a role in several human diseases (76, 77), and have become promising candidates for rational drug design efforts. Thus, the prediction of IDR in these novel spliced isoforms is a critical component of the effective drug design. The IDR prediction architectures are divided into three categories:

1. Sequence scoring functions, using the additive and weighted functions to process the input protein sequences and sequence-derived information, such as FoldIndex (78) and IUPred3 (79).
2. Machine learning-based models trained from experimentally characterized IDRs, such as DisEMBL (80), DISOPRED3 (81), SPOT-Disorder2 (82), and flDPnn (83).
3. Meta-predictors, using multiple disorder predictions as inputs to generate the new disorder prediction, such as metaPrDOS (84), MobiDB-lite (85), and DisCoP (86).

Additionally, conserved binding motifs are often coded in tissue-specific alternatively spliced exons, which results in the tissue-specific rewiring of protein-protein interaction networks (74, 87). More recent work has suggested the potential for these protein-protein interactions to drive oncogenic transformation (5). Consequently, understanding the protein-rewiring by alternatively spliced membrane receptors, such as G protein-coupled receptors (GPCRs) (88–90), a major class of druggable receptors for cancer therapy (91), will be critical for future drug design efforts. GPCRs are a superfamily of proteins affected by various post-translational modifications (92) and alternative splicing (93) and are recognized for their role in cancer development, progression, and metastasis (94–96). Receptors, such as GPCRs, undergo significant conformational changes upon binding to their targets, which add to the structural and functional heterogeneity of their disordered regions, further intensified by cancer-related mutations (96).** **For example, the unique isoforms and their IDRs (detected mainly in the 3^rd^ intracellular loop and the C-terminal tail) are typically enriched in tissue-specific isoforms, resulting in tissue-specific protein-protein interactions  (90), including the GPCRs  (97). Notably, the IDRs in the C-terminal of GPCRs are proposed to have a high potential for new target-binding partners (98). The importance of the N-terminal for the activation of two distinct GPCR isoforms was also highlighted (99). Given the richness of the conformational rearrangements and expression patterns across tissues, the GPCR isoforms represent new targets for developing drugs with improved tissue selectivity (93). Currently, there are a number of computational algorithms for modeling GPCR structure and ligand binding sites and their binding affinities, such as GPCR-I-TASSER (100), Computational Profiling for GPCRs (CPG) (101), GPCR_LigandClassify (102), and an unnamed method by Seo et al. (103), and conformation-specific thermostabilizing mutations predictive tool LiticonDesign (104). GPCR models include PRECOG (105), a machine learning algorithm that can model the coupling of GPCR isoforms and the G-protein, which can be leveraged as a target by Designer Receptors Exclusively Activated by Designer Drugs (106). Regardless, algorithms that fully consider the GPCR proteoform diversity is limited and remains a major knowledge gap in the field of GPCR drug discovery.

Another functional impact of alternative splicing is its effect on post-translational modification (107). PTMs are widely distributed in the protein universe, which include phosphorylation, ubiquitination, SUMOylation, lipidation, and glycosylation. These PTMs influence protein function by regulating protein cleavage, linkage, and cross-linking. Understanding the splicing impact on PTMs will enhance our ability to prioritize targetable protein regions. For example, splice variants that result in drug resistance include, BCL-ABL (108), FGFR (109), and HER2 (110). In recent years, several computational methods have been developed to predict PTMs in a whole protein sequence, such as MusiteDeep (111), iAcet-Sumo (112), GPS 5.0 (113). Although several high-throughput methods have been developed to identify the PTMs, this remains challenging and experimental validation of the PTMs by mass spectrometry is necessary to confirm the computational predictions. Therefore, a joint multi-omics profiling of splicing events and its post-translational signals will be critical in guiding future directions in targeted therapy, especially oncogenic kinase signaling. An alternative PTM modification is by glycolipid on glycosylphosphatidylinositol (GPI) anchor proteins. GPI-anchor proteins are membrane proteins and link to proteins to the outer face of the plasma membrane in eukaryotic cells, making them ideal targets for therapeutic intervention, including CAR T cell engineering. For example, GPI-anchor protein under consideration by CAR T cell include CEA (114), GPC1 (115), GPC2 (116), GPC3 (117), FOLR2 (118), and Mesothelin (119). While several predictors have been designed specifically for GPI-anchor predictions, such as GPI-SOM (120), FragAnchor (121), and PredGPI (122), no studies have performed a systematic analysis of cancer-specific protein isoform that may have gained a novel GPI-anchor. Thus, this represents a tremendous opportunity to uncover additional targets for therapeutic targeting.

## Discussion

Alternative splicing is one of the major contributors to protein variation and diversity, which provide cancer cells the cellular plasticity to survive stress (123). With the availability of CPTAC data, emerging studies are investigating a multi-omics approach for integrated splice variants discovery but are limited to the purpose of disease monitoring and prognosis determination (124, 125). Here, our scientific predicate (in this review) is that cancer-specific alternative splicing event is an untapped source of targets for therapeutic intervention, and a complete multi-omics characterization will enable the discovery, validation, and prioritization of the ASE targets. 1) we propose that the joint analysis of long and short RNA sequencing will enable a comprehensive search for novel splice variants in which long RNA sequencing is more suited for identifying novel isoforms, and short-read RNA sequencing will be able to estimate transcript abundance. Genotype-Tissue Expression (GTEx) project (60, 126, 127) can also be utilized as a normal tissue reference for the therapeutic discovery process. Significant multi-omics data has been generated by the GTEx community, which includes short-read RNA sequencing (17364 samples), Oxford Nanopore Long-read sequencing (88 samples), and TMT mass spectrometry proteomics data (32 samples). Jointly these data will provide a valuable normal control that enhances the therapeutic candidate selection process of cancer-restricted isoform variant while reducing unwanted adverse effects during therapy. 2) we propose that proteogenomic integration of RNA seq and mass spectrometry will enable the validation of the protein product derived from the aberrant splicing event. 3) with tremendous therapeutic interest in immunotherapy, kinase inhibition, and GPCR drug response, we propose that a structural understanding of gaining and losing PTM and IDR domains by alternative splicing will be necessary to prioritize important proteoforms with the spliced exon. Thus, protein structure annotation followed by mass spectrometry validation will enable the joint prediction and validation of new post-translational modification sites on the novel proteoform. Together, the development of these new technologies and integrative pipelines will enable us to leverage multi-omics data for therapeutic discovery.

Several challenges in each omics need to be considered in future development. 1) for both long read sequencing and mass spectrometry analysis, obtaining deep coverage remains costly in terms of time and money. Additionally, technical challenges with coverage bias will also need to be addressed. To overcome this issue, the harmonization of different datasets may enhance our ability to discover novel targets. The assumption is a recurring candidate, while suboptimal for detection in certain settings, can then be rescreened by informatics processing or through a targeted mass spectrometry or re-sequencing effort. 2) algorithm prediction of proteoform function will need to be validated by experiments. While we have proposed that mass spectrometry data may be one mechanism to validate the PTMs, the oncogenic role of the region remains to be validated. For trans-acting variants in splicing factors, such as mutations in SF3B1, a genetic mouse model will be necessary to evaluate its effect on malignant transformation (128). For cis-acting variants and therapeutic vulnerabilities, high-throughput functional screening of alternative splicing events, such as CRISPR (129) and shRNA (130), can be performed in different cancer cell lines. These models will evaluate phenotypes associated with proliferative gains or cancer-killing effects in the presence of other oncogenic drivers. Of note, current high-throughput genetic screening of adult cancers DepMap (131), Sanger (132), as well as in pediatric cancer (133) are restricted to gene-level knockouts. Thus, to fully comprehend the functional role of splice variants in cancer, high throughput CRISPR and shRNA libraries that target junctions will need to be developed in future studies.

Cancer-derived isoforms remains a dark matter in the context of cancer therapy. New omics dataset and structure prediction strategies will be critical for understanding the splicing function as well as optimizing therapeutic development priorities. Additional technologies, such as machine learning, will be essential to automate the integrative discovery platform from RNA-to-Protein-to-Structure-to-Function. In summary, the complete integration of these omics technologies will surely be necessary to facilitate the next generation of small molecule design, immunotherapeutic development, and to overcome therapeutic relapse and drug resistance.

## Author contributions

TS, BZ, YL, HW, LW, BM, PS, and AK contributed to the writing and review of the manuscript. TS, PS, and AK contributed to the conception of the review paper. All authors contributed to the article and approved the submitted version.

## Funding

The study was funded by American Cancer Society IRG-21-145-25, Moffitt Cancer Center Quantitative Science/Team Science, Moffitt Cancer Center Department of Biostatistics and Bioinformatics Pilot Award. This work has been supported in part by the Biostatistics and Bioinformatics Shared Resource at the H. Lee Moffitt Cancer Center & Research Institute, an NCI designated Comprehensive Cancer Center (P30-CA076292).

## Conflict of interest

TS reports a patent for EBD CAR US20220267425A1.

The remaining authors declare that the research was conducted in the absence of any commercial or financial relationships that could be construed as a potential conflict of interest.

## Publisher’s note

All claims expressed in this article are solely those of the authors and do not necessarily represent those of their affiliated organizations, or those of the publisher, the editors and the reviewers. Any product that may be evaluated in this article, or claim that may be made by its manufacturer, is not guaranteed or endorsed by the publisher.
